# Long- and short-term acclimation of the photosynthetic apparatus to salinity in *Chlamydomonas reinhardtii*. The role of Stt7 protein kinase

**DOI:** 10.3389/fpls.2023.1051711

**Published:** 2023-04-05

**Authors:** Elsinraju Devadasu, Sai Divya Kanna, Satyabala Neelam, Ranay Mohan Yadav, Srilatha Nama, Parveen Akhtar, Tamás F. Polgár, Bettina Ughy, Győző Garab, Petar H. Lambrev, Rajagopal Subramanyam

**Affiliations:** ^1^ Department of Plant Sciences, School of Life Sciences, University of Hyderabad, Hyderabad, India; ^2^ Institute of Plant Biology, Biological Research Centre, Eötvös Loránd Research Network, Szeged, Hungary; ^3^ Doctoral School of Biology, University of Szeged, Szeged, Hungary; ^4^ Department of Biochemistry, School of Life Sciences, University of Hyderabad, Hyderabad, India; ^5^ Institute of Biophysics, Biological Research Centre, Eötvös Loránd Research Network, Szeged, Hungary; ^6^ Theoretical Medicine Doctoral School, University of Szeged, Szeged, Hungary; ^7^ Department of Physics, Faculty of Science, University of Ostrava, Ostrava, Czechia

**Keywords:** acclimation, *Chlamydomas reinhardtii*, macroorganization, photosystems 1 and 2, salt stress, state transitions of photosynthetic apparatus, Stt7 kinase, thylakoid membrane

## Abstract

Salt stress triggers an Stt7-mediated LHCII-phosphorylation signaling mechanism similar to light-induced state transitions. However, phosphorylated LHCII, after detaching from PSII, does not attach to PSI but self-aggregates instead. Salt is a major stress factor in the growth of algae and plants. Here, our study mainly focuses on the organization of the photosynthetic apparatus to the long-term responses of *Chlamydomonas reinhardtii* to elevated NaCl concentrations. We analyzed the physiological effects of salt treatment at a cellular, membrane, and protein level by microscopy, protein profile analyses, transcripts, circular dichroism spectroscopy, chlorophyll fluorescence transients, and steady-state and time-resolved fluorescence spectroscopy. We have ascertained that cells that were grown in high-salinity medium form palmelloids sphere-shaped colonies, where daughter cells with curtailed flagella are enclosed within the mother cell walls. Palmelloid formation depends on the presence of a cell wall, as it was not observed in a cell-wall-less mutant CC-503. Using the *stt7* mutant cells, we show Stt7 kinase-dependent phosphorylation of light-harvesting complex II (LHCII) in both short- and long-term treatments of various NaCl concentrations—demonstrating NaCl-induced state transitions that are similar to light-induced state transitions. The grana thylakoids were less appressed (with higher repeat distances), and cells grown in 150 mM NaCl showed disordered structures that formed diffuse boundaries with the flanking stroma lamellae. PSII core proteins were more prone to damage than PSI. At high salt concentrations (100–150 mM), LHCII aggregates accumulated in the thylakoid membranes. Low-temperature and time-resolved fluorescence spectroscopy indicated that the *stt7* mutant was more sensitive to salt stress, suggesting that LHCII phosphorylation has a role in the acclimation and protection of the photosynthetic apparatus.

## Introduction

Salinity is one of the most significant risks to the agricultural sector. Acid rains trigger the release of sodium and bicarbonate ions into the environment; salts dispersed through sewage, fertilizers used on farms, and the salting of roads cause the increased salinity of streams, rivers, and lakes (Lockwood, 2019). Approximately 20% of the world’s cultivated land and approximately 50% of the irrigated lands are affected by salinity (Measho et al., 2022). Salinity imparts both ionic and osmotic stresses as primary effects, which may further lead to secondary impacts like oxidative damage resulting in loss of membrane integrity, enzyme activity, reduced nutrient acquisition, and photosynthetic efficiency. Additionally, a high concentration of salts infringes on ion homeostasis and leads to molecular damage, growth arrest, and ultimately death if the organism fails to adapt to the salinity stress.

It is well-known that high salt concentrations affect photosynthesis and plant productivity. Previous reports showed that the exposition of photosystem (PS)II particles to higher NaCl concentrations resulted in the separation of extrinsic proteins of the oxygen-evolving complex (OEC), leading to impaired oxygen evolution (Murata and Miyao, 1985). Salt stress inhibited PSII activity in mangrove trees (Tiwari et al., 1998; Parida et al., 2003). In addition, NaCl inhibited the electron transport at the donor and acceptor sides of PSII in cyanobacteria (Verma and Mohanty, 2000). Earlier studies reported that both PSI and PSII were inactivated by osmotic stress (Allakhverdiev et al., 2000; Al-Taweel et al., 2007). Reports showed that salt could affect the protein levels in the photosynthetic apparatus (Parida et al., 2003; Sudhir et al., 2005; Subramanyam et al., 2010; Neelam and Subramanyam, 2013), but the organization of photosynthetic complexes and the role of plastoquinone (PQ) pool are not known.

The readjustment of absorption cross-sections of PSII and PSI can occur in both short- and long-term periods (by transcriptional and translational regulation of light-harvesting complexes (LHC) gene expression). In the short term (seconds–minutes), light energy balance is sensed through, e.g., ΔpH changes across the membrane and the PQ pool’s redox state, which activates non-photochemical quenching and state transitions, respectively. On a longer timescale, the composition of light-harvesting systems of both the photosystems, PSII and PSI, varies.

The PQ pool is part of a complex signaling network consisting of cytochrome (Cyt) *b*_6_/*f*, plastocyanin, protein kinases, and phosphatases that all together work collectively to restore the redox poise of the electron carriers to maintain the photosynthetic efficiency (Cruz et al., 2001; Borisova-Mubarakshina et al., 2015). Over-excitation of PSII relative to PSI results in the reduction in the PQ pool and binding of PQH_2_ to the Qo site of Cyt *b*_6_/*f*, which further activates Stt7 (state transition kinase 7) or STN7, phosphorylating a portion of LHCII (Depège et al., 2003; Bellafiore et al., 2005; Minagawa, 2011). This shifts the system into State II. The process is reversed when the PQ pool is oxidized, which results in the inactivation of the kinase, dephosphorylation of LHCII by the PPH1/TAP38 phosphatase, and its return to PSII, reverting to State I (Pribil et al., 2010; Shapiguzov et al., 2010). In State II conditions, *C. reinhardtii* forms large PSI–LHCI–LHCII supercomplexes that contain 9 or 10 LHCI subunits, 2 LHCII trimers, and possibly a monomeric LHCII subunit CP29 (Drop et al., 2014; Huang et al., 2021).

The absorption cross-section of the PSI and PSII can be modulated by anaerobiosis (Nellaepalli et al., 2012) and elevated temperature (Nellaepalli et al., 2011; Nellaepalli et al., 2015; Madireddi et al., 2019). Anaerobiosis-induced state transition was observed in *Arabidopsis thaliana*, which also mimics the light-induced state transitions. Dark anaerobiosis shows a non-photochemical reduction in the PQ pool due to respirational deprivation and depleted ATP content. Where the reduction in the PQ pool is proposed to be mediated by an NDH-dependent pathway, it was reported that the absence of O_2_ negatively affects the activity of PTOX, which fails to oxidize the PQ pool (Cournac et al., 2000). This leads to the activation of LHCII kinase, which phosphorylates LHCII—resulting in the migration of LHCII complexes to PSI (Nellaepalli et al., 2012). Non-photochemical reduction in the PQ pool can occur by anaerobiosis (Finazzi et al., 2002; Nellaepalli et al., 2012) and heat (Havaux, 1996; Bukhov and Carpentier, 2004; Nellaepalli et al., 2011).

The impact of moderately elevated temperature on state transitions is well documented (Nellaepalli et al., 2011; Nellaepalli et al., 2015; Madireddi et al., 2019). Dark, elevated temperatures up to 40°C can induce LHCII phosphorylation; thus, state transitions occur in these conditions. With the combination of dark and high temperatures, the reduction in the PQ pool is driven by an increase in thylakoid membrane fluidity and leakiness. Predominantly PGR5-independent cyclic electron transfer is responsible for inducing non-photochemical reduction in the PQ pool under moderately elevated temperatures from *A. thaliana*. Even the PQ pool is reduced without light, which means that the source of electrons can be from enhanced malate and glycolytic pathway (Bennoun, 1982; Nellaepalli et al., 2012). In *Chlamydomonas (C.) reinhardtii*, thylakoid membrane dynamics could have altered under elevated temperature due to a change in membrane fluidity. The state transition complexes separated using sucrose density centrifugation from *C. reinhardtii* under elevated temperature have shown the presence of a PSII–LHCII–PSI complex that can redistribute energy between two photosystems within the same complex (Madireddi et al., 2019). In all these reports, the dark-induced state transition may be involved in photoprotection and balance of the excitation pressure on both photosystems.

In this study, we investigated the long- and short-term acclimation responses of *C. reinhardtii* to NaCl, specifically clarifying the role of LHCII and the redox-activated chloroplast protein kinase Stt7 (state transition kinase 7) in mediating the acclimation response.

## Experimental procedures

### Growth conditions

In this study, we used wild-type strain of *C. reinhardtii* (CC-125) and mutant state transition kinase 7 (*stt7)* (a kind gift from Prof. Yuchiro Takahashi, Okayama University, Japan), Cell-wall-deficient mutant (CC)-503 (obtained from the *Chlamydomonas* resource center, University of Minnesota). Cells were grown to mid-log phase in Tris–acetate–phosphate (TAP) medium at 25°C under cool fluorescent light (50 μmol photons m^−2^ s^−1^) as reported earlier (Neelam and Subramanyam, 2013). The cells grown in 50 μmol photons m^−2^ s^−1^ always showed that the PQ pool is oxidized as reported earlier (Depège et al., 2003; Bellafiore et al., 2005; Takahashi et al., 2006). However, in some cases, the *pbcb* mutant of *C. reinhardtii* can phosphorylate in low and high light, which is different from the wt (Cariti et al., 2020).

WT CC-125, *stt7*, and CC-503 cells were grown in various sodium chloride (NaCl) concentrations (50, 100, and 150 mM NaCl). WT CC-125 cells were grown under control (NaCl-free) and 150 mM NaCl until cells reached the mid-log phase. Continuous growth measurements of control and salt-stressed cells were performed daily, and Chl concentration and Chl *a*/*b* ratio were determined (Porra et al., 1989). For all parameters, the mid-log cells were used.

For short-term phosphorylation studies, we have treated the mid-log phase cells of WT CC-125 and *stt7* strains with various salt concentrations (50, 100, 150, and 200 mM NaCl) for 20 min.

### Isolation of thylakoid membranes

Cells from respective salt treatments were harvested for isolation of thylakoid membranes as described earlier (Fischer et al., 1997); resuspended in buffer containing 200 mM sorbitol, 5 mM Tris–HCl (pH 7.5), 2 mM NaF, proteolytic inhibitor (1 mM benzamidine HCl and 1 mM amino caproic acid), 10 mM MgCl_2_, and 5 mM CaCl_2_; and stored at −80°C for further experiments.

### Confocal microscopy

*C. reinhardtii* cells of WT CC-125 and CC-503 grown in various salts, namely, sodium chloride (NaCl), potassium acetate (CH_3_COOK), and potassium chloride (KCl) with different concentrations (0, 50, 100, and 150 mM), were immobilized with equal amounts of molten agar at room temperature. Images were captured using a Leica confocal microscope, and the cells were viewed with a 63× oil immersion lens objective using the ZEN 2010 software.

### Measurements of oxygen evolution and uptake

Oxygen measurements were performed with an oxygen electrode (Hansatech, UK) at 25°C. Oxygen evolution activity was measured with WT CC-125 cells grown in 0 and 150 mM conditions using phenyl-p-benzoquinone (0.5 mM) as an electron acceptor. The Chl concentration of the samples was 10 μg Chl/ml. PSI-mediated electron transfer from reduced dichlorophenol indophenol (DCPIPH_2_) to MV was measured by light-induced O_2_ consumption. The reaction mixture is composed of freshly isolated thylakoids (15 μg Chl/ml), 20 mM Tricine–KOH (pH 8.0), 0.5 mM MV, 1 mM NaN_2_, 0.1 mM DCPIP, 5 mM ascorbate, and 10 μM DCMU. Samples were incubated in the dark for 5 min before measurement.

### Transmission electron micrograph measurement

Approximately 0.5 ml of control and salt-treated cell samples from all groups (*n* = 2/group) of both WT and *stt7* mutant were immersed into a modified Karnovsky fixative solution (Darnovsky, 1965) (pH 7.4), which contained 2% paraformaldehyde (Sigma-Aldrich; St. Louis, MO, USA) and 2.5% glutaraldehyde (Polysciences; Warrington PA, USA) in phosphate buffer. Samples were fixed overnight at 4°C, then briefly rinsed in distilled water (pH 7.4) for 10 min and fixed further in 2% osmium tetroxide (Sigma-Aldrich, St. Louis, MO, USA) in distilled water (pH 7.4) for 2 h. After osmification, samples were briefly rinsed in distilled water for 10 min, then dehydrated using a graded series of ethanol (Molar Chemicals; Halasztelek, Hungary), from 50% to 100% for 30 min in each concentration. Afterwards, cells were processed through propylene oxide (Molar Chemicals) and embedded in an epoxy-based resin (Durcupan ACM; Sigma-Aldrich). After polymerization for 48 h at 56°C, resin blocks were etched, and 50-nm thick ultrathin sections were cut using an Ultracut UCT ultramicrotome (Leica; Wetzlar, Germany). Sections were mounted on a single-hole, formvar-coated copper grid (Electron Microscopy Sciences; Hatfield, PA, USA), and the contrast of the samples was enhanced by staining with 2% uranyl acetate in 50% ethanol (Molar Chemicals, Electron Microscopy Sciences) and 2% lead citrate in distilled water (Electron Microscopy Sciences) (Reynolds, 1963; Hayat, 1970).

Ultrathin sections from the cells were screened at 3,000× magnification with a JEM‐1400 Flash transmission electron microscope (JEOL; Tokyo, Japan) until 25–30-granum cross-sections were identified from each sample (n = 2/group). For quantitative measurements of the repeat distance, images of thylakoid membrane structures were recorded at 50,000× magnification using a 2 k × 2 k Matataki Flash scientific complementary metal‐oxide‐semiconductor camera (JEOL). Finally, quantitative analysis of the membrane repeat distances was performed using the built-in measurement module of the microscope.

Repeat distances were measured from the control and salt (100 mM NaCl)-treated cells of WT and *stt7* in two sets (biological repetitions). Some 40 repeat distances were measured from each group by choosing 10 cells (four measurements) from each group. All the repeat distances were averaged, and the average of the biological repetitions was averaged as indicated in **Supplementary Table S1**.

### RNA extraction and gene expression studies

*C. reinhardtii* cultures (OD_750_ = 0.8) from the above conditions were harvested by centrifugation (10 min at 4,500 rpm, 4°C). Total RNA extraction was performed using the RNeasy Plant Mini Kit (Sigma, St. Louis, MO, USA). Briefly, the cell pellet from the 5-ml culture was resuspended in 400 µl lysis buffer RLT and frozen in liquid nitrogen for 3 min. Cells were then lysed by incubating them for 2 min at 60°C. The integrity of the RNA was checked by electrophoresis on 2% denaturing agarose gels. RNA quality was assessed by determining the 260 nm/280 nm absorbance ratio using a Nanodrop ND-1000 instrument (Thermo Scientific, Germany). DNase treatment was given using RNase-Free DNase Set (Qiagen-79254). RNA from each sample was used for cDNA synthesis (Bio-Rad) and amplified using the SYBR Green PCR Master Mix. Reverse Transcriptase Reagents (Bio-Rad), following the one-step RT-PCR protocol recommended by the manufacturer. The design of primer pairs was based on the *C. reinhardtii* gene models of the genome annotation v.5.5 released by the Joint Genome Institute (DOE/JGI; https://phytozome.jgi.doe.gov/pz/portal.html). PCRs were performed in the Bio-Rad CFX 96 real-time system for signal detection. A negative control without the template was used to assess the overall specificity. Histone H3 was used as the endogenous control for calculating the relative abundance of each gene. Each assay was triplicated. Primer design and their optimization regarding primer dimerization, self-priming formation, and primer melting temperature (melting temperature of 59°C–60°C and product sizes between 100 and 160 bp) were done using a primer analysis software, IDT Oligo Analyzer Tool (https://www.idtdna.com/pages/tools/oligoanalyzer). The list of primers of each gene with their gene ID was made (**Supplementary Table S2**). The relative abundance of each gene was expressed relative to the reference and calculated as ^ΔΔ^CT values.

### 2D blue-native PAGE

Thylakoids containing an equal amount of Chl (30 μg) were resuspended in ACA buffer (750 mM ϵ-aminocaproic acid; 50 mM Bis-Tris, pH 7.0; 0.5 mM EDTA) and solubilized by the addition of β-dodecyl maltoside to a final concentration of 2% on ice for 20 min. The unsolubilized material was removed by centrifugation at 12,000 rpm for 10 min at 4°C. The supernatant was mixed with loading dye (750 mM ϵ-aminocaproic acid and 5% Coomassie Brilliant Blue G 250) in a 1:1 ratio and loaded onto each lane. Blue native electrophoresis was performed as described earlier, albeit with some modifications (Schägger and von Jagow, 1991).

For the second dimension, each lane was dissected carefully, separated from the 1D blue native gel, and kept for solubilization in a buffer (66 mM Na_2_CO_3_, 100 mM β-mercaptoethanol, and 2% SDS) for 20 min and loaded onto a 12% Tricine SDS-PAGE gel containing 2 M urea (Ossenbühl et al., 2004). The 2D-BN/SDS gels were ran carefully until the dye front reached the end of the gel. The gels were stained with Coomassie brilliant blue.

### Western blotting

Thylakoid membranes were separated using SDS-PAGE. Equal quantities of Chl (2 μg) or protein (20 µg) were loaded onto each lane. To analyze and appraise quantitatively the polypeptide content in the thylakoids, immunoblotting was performed as described by Towbin and coworkers (Towbin et al., 1979). Proteins were electrophoretically transferred onto polyvinylidene fluoride (PVDF) membranes. The membrane was incubated with polyclonal antibodies raised in rabbits. We have used the specific primary antibodies against PSI core (PsaA, PsaB, PsaC, PsaD, PsaG, and PsaH), Lhcb proteins (Lhcb1, Lhcb2, Lhcb4, Lhcb5, and Lhcbm5), PSII core (PsbA, PsbC, PsbD, PsbO, and PsbP) (all purchased from AGRISERA, Vännäs, Sweden, www.agrisera.com), and Lhca proteins (Lhca1, Lhca2, Lhca3, Lhca4, Lhca5, Lhca6, Lhca7, and Lhca9). Peptide tag antibodies for Lhca complexes were developed in our laboratory (Yadavalli et al., 2012). Subsequently, secondary antibodies ligated to horseradish peroxidase (HRP) were applied. We performed 2D-BN-SDS-Western blotting with respective antibodies to test the protein content changes due to salt treatment. After salt treatment, an anti-phosphothreonine antibody was used to reveal phosphorylated threonine residues in PSII proteins. Chemiluminescence reagents were used to develop the PVDF membrane. The images were recorded with a Bio-Rad CCD camera.

### Fluorescence spectroscopy

#### Steady-state fluorescence

Low temperature (77 K) fluorescence spectra of *C. reinhardtii* cells (WT, *stt7*) grown in various salt concentrations (0, 50, 100, and 150 mM) were recorded with a Jobin Yvon Fluorolog spectrofluorometer. The cell suspensions equivalent to 5 µg Chl/ml were filtered and deposited on Whatman glass fiber (GF/B) discs. The filters were frozen in liquid nitrogen and transferred to a liquid nitrogen-filled Dewar vessel placed into the measurement chamber. Chl *a* was excited at 436 nm, and a bandwidth of 3 nm was used for recording the emission spectra from 650 to 800 nm.

#### Time-resolved fluorescence

Time-correlated single-photon counting measurements were performed at room temperature using standard protocols (Akhtar et al., 2016). *C. reinhardtii* cells (WT, *stt7*) grown in various salt concentrations (0, 50, 100, and 150 mM NaCl) were excited with 633-nm pulses of 6 ps at a repetition rate of 20 MHz. The instrument response function (IRF) was obtained (40 ps) with 5% Ludox as a scattering solution. Fluorescence emission data were recorded between 670 and 750 nm, binned in 4 ps time channels. Data analysis was performed by a global lifetime fitting routine using a kinetic model and iterative re-convolution of the simulated decays with the measured IRF. The fitting algorithm, written in MATLAB, minimized the square sum of residuals weighted by the Poisson distribution.

### Chlorophyll *a* fluorescence induction kinetics

Chl *a* fluorescence (O–J–I–P) transients were recorded at room temperature with a PEA fluorometer (Hansatech, UK). All samples (both long- and short-term NaCl treated) were dark adapted for 10 min before measurement. The actinic light intensity reaching the cells was 1,500 μmol photons m^−2^ s^−1^. Biolyzer HP3 (Fluoromatics, Switzerland) software was used to calculate and visualize O–J–I–P parameters quantifying the energy through PSII.

### Circular dichroism spectroscopy

Circular dichroism (CD) spectra of cells from *C. reinhardtii* (control and salt grown) WT and *stt7* strains were recorded using a JASCO-815 spectropolarimeter at room temperature. The cells were diluted to a Chl concentration of 20 µg/ml for each sample. For measurements in the visible region (400–800 nm), a standard glass cuvette with a path length of 1 cm was used. Three scans were accumulated with continuous scan mode and a scan speed of 100 nm min^−1^, with data collected every nanometer. Spectra were baseline corrected using a TAP medium.

### Determination of protein aggregates in the insoluble fraction

To determine the aggregated proteins in the pellet, thylakoids were isolated from the salt-treated cells, and the isolated thylakoid membranes were solubilized in 1% n-dodecyl β-D-maltoside (β-DM). The reaction mixture was incubated in the dark for 10 min and centrifuged at 10,000×*g* for 10 min at 4°C. The final Chl concentration of 0.8 mg ml^−1^ Chl was maintained in both control and salt-treated samples. The flow-through was discarded, and the pellet was dissolved in a solubilization buffer containing 2% β-mercaptoethanol. We took 10 μl of protein and loaded it onto the gel. After running the electrophoresis, the gel was transferred to a nitrocellulose membrane using a semi-dry transfer blot. We have immunoblotted the Lhcb2 and Lhcbm5 antibodies to see the protein abundance in the aggregation part.

## Results

### Salt-induced palmelloid formation

**Figure 1:**
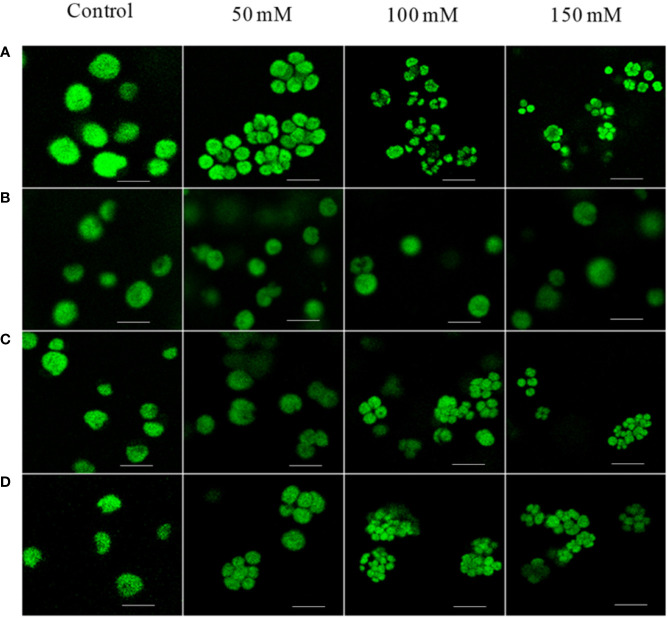
Confocal microscopy images of *C. reinhardtii* strains: WT, cell-wall mutant CC-503 grown in media with different NaCl concentrations (50, 100, and 150 mM; control, no addition). **(A)** WT strain showing palmelloid formation under all NaCl concentrations, **(B)** cell-wall-mutant (CC)-503 strain; palmelloid formation was absent under all NaCl concentrations. Palmelloid formation in the WT strain was noticed in **(C)** from 50 to 150 mM potassium acetate (CH_3_COOK), and **(D)** from 50–150 mM potassium chloride (KCl) concentrations. A scale bar of 10 µm was maintained for all the images.

We have previously reported the formation of palmelloids in *C. reinhardtii* cells grown in high-salt conditions (Neelam and Subramanyam, 2013). Cells, generally free-living, enter a transient colony-like stage called a “palmelloid,” during which they exhibit distinctive physiological and morphological characteristics. Palmelloid formation is associated with abnormal cell wall production and loss or impairment of flagellar function; hence, we hypothesized that palmelloid formation under salt stress resulted from cell wall abnormality. To verify our hypothesis, we chose a CC-503 class III cell wall mutant, which releases its cell wall into the medium. We imaged the Chl fluorescence of CC-503 cells grown in different salts (potassium chloride) along with sodium chloride (NaCl) containing media (0, 50, 100, and 150 mM) by confocal microscopy. The images showed CC-503 mutant cells growing individually, without aggregating, in contrast to WT control cells (**Figure 1**). The salt-tolerant CC-503 strain grows two times faster than the WT CC-125 (data not shown). We also checked the palmelloid-inducing ability of other salts, potassium acetate (CH_3_COOK) and potassium chloride (KCl). As expected, these salts also induced palmelloid formation in *C. reinhardtii* (**Figure 1**). However, with increasing NaCl concentration (50, 100, and 150 mM), the total Chl content was strongly reduced after 4 days, compared to the 0-day control in WT and *stt7* (state transition kinase 7), particularly in cells grown in 100 and 150 mM NaCl (**Supplementary Figure S1A**).

### PSII and PSI activity

The PSII activity, oxygen evolution, was drastically reduced in cells grown in media containing 150 mM NaCl (**Supplementary Figure S1B**). Furthermore, PSI activity was monitored by measuring the oxygen uptake in the presence of methyl viologen (MV), showing a significant increase in PSI activity (**Supplementary Figure S1C**). The growth rate is following the rate of oxygen evolution (**Supplementary Figure S1D**). In line with the oxygen evolution results, fast chlorophyll *a* fluorescence induction curves (OJIP transients, **Supplementary Figure S2A**) showed an increase in the initial fluorescence level (F_o_). The normalized spectra of Chl *a* fluorescence at “O” and “P” levels were similar under high salt than the control in WT; however, both these levels were severely declined in *stt7* in the control condition (**Supplementary Figure S2B**). In the cells grown in 150 mM NaCl, the maximum fluorescence level (F_m_) and the photosynthetic activity (F_v_/F_m_) were decreased (**Supplementary Figure S2C**), which correlated with the decrease in growth rate (**Supplementary Figure S1D**). However, the WT is less affected by salt than *stt7*. Short-term (20 min) treatment of cells with NaCl also led to a noticeably lower F_m_ (and F_v_/F_m_), showing the rapid inactivation of PSII.

### Steady-state fluorescence spectra

**Figure 2:**
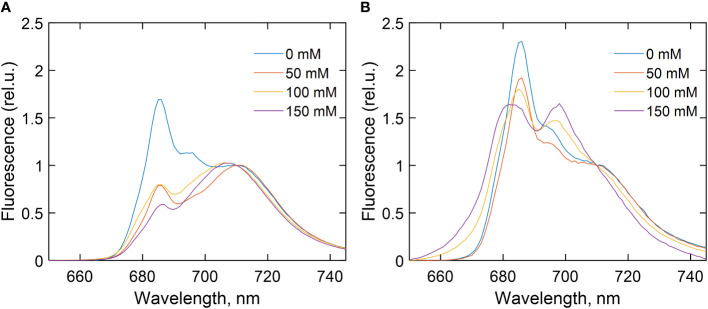
77 K fluorescence emission spectra of **(A)** wild type (WT), **(B)** state transition kinase 7 (*stt7)* strains of *C. reinhardtii* cells grown in media containing different concentrations of NaCl as indicated. The spectra are averages from at least three measurements on different batches and are normalized at 710 nm. For further details, see experimental procedures.

**Figure 3:**
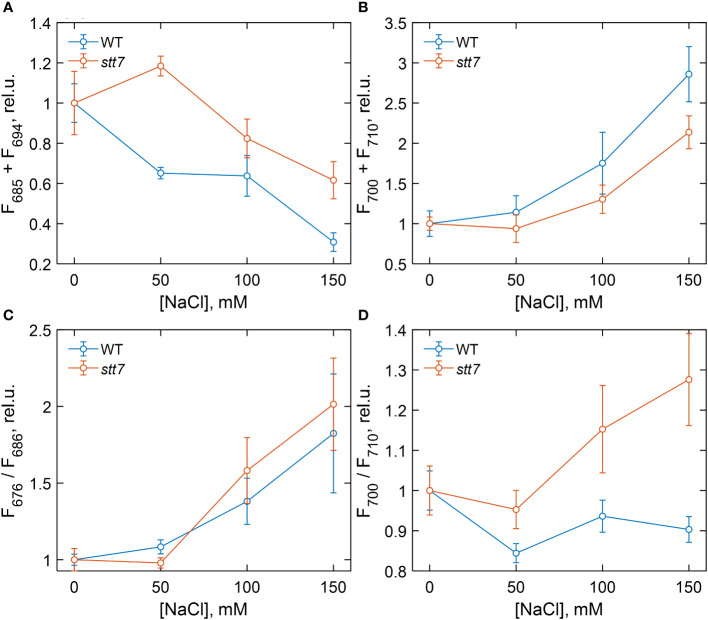
Dependence of different 77 K fluorescence emission parameters of wild-type (WT) and state transition kinase 7 (*stt7)* cells on the sodium chloride (NaCl) concentration of growth medium, relative to the salt-free control group. **(A)** Sum of F685 and F694; **(B)** sum of F700 and F710; **(C)** F676 relative to F686; **(D)** F700 relative to F710. The values are calculated from the areas of the corresponding Gaussian components normalized to the total area under the fluorescence curves (except for F676, which is normalized to F685) and plotted relative to the corresponding value in the control. Symbols and bars indicate mean and standard error, n = 3–6. The closed symbols represent statistically significant differences (p < 0.05) with the respective control (0 mM), based on multiple comparison ANOVA.

To evaluate the excitation energy distribution and changes in the absorption cross-section of the two photosystems during salt stress, fluorescence emission spectra were recorded at 77 K from *C. reinhardtii* cells WT (**Figure 2A**) and *stt7* (**Figure 2B**) grown at different salt concentrations (0, 50, 100, and 150 mM NaCl) (**Figure 2**). Averaged spectra normalized to their intensity at 710 nm are plotted in **Figure 2**. The emission bands at 685 and 694 nm originate from the PSII reaction center (RC) and PSII core antenna (CP43 and CP47), respectively, whereas the 715-nm band arises from PSI (Murata et al., 1966). In all *C. reinhardtii* strains grown in high-salinity media, the relative intensity of the PSII emission bands was significantly reduced. In addition, at 100 mM NaCl or higher, the spectra showed an apparent blue shift of the far-red maximum, which could be attributed to a relative diminishment of the PSI emission band and the appearance of an additional band around 700 nm. The latter could be interpreted as originating from aggregated LHCII trimers (Andreeva et al., 2003; Kirchhoff et al., 2003). In parallel, a shoulder in the 670–680-nm range indicated the presence of uncoupled LHCs, and, in some cases, increased emission below 670 nm signified the presence of Chls that were not bound to the protein complexes. These effects appeared more clearly in *stt7* cells grown in 150 mM NaCl condition (**Figure 3**).

For a quantitative description of the observed spectral changes, the fluorescence spectra were subjected to Gaussian decomposition by non-linear least-squares fitting. The spectra of WT cells grown in the control medium were modeled with four Gaussian bands centered at 678, 685, 694, and 710 nm (**Supplementary Figure S3A**). An additional band peaking at 700 nm, ascribed to aggregated LHCII, was present in salt-treated cells (**Supplementary Figure S3B**). The relative areas of the fluorescence components associated with PSII (F685 + F694) and with PSI and aggregates (F700 + F710) are plotted in **Figures 3A, B**, respectively, in terms of mean values and standard errors from independent experiments. **Figure 3** also shows the fluorescence intensity ratios F676/F686, representing the amount of free LHCs (**Figure 3C**), and F700/F710, reflecting aggregated LHCs (**Figure 3D**). Analysis of variance (one-way ANOVA) showed that high salinity significantly affected all four fluorescence parameters (**Supplementary Table S3**). In WT cells, the relative PSII emission intensity gradually decreased with increasing salinity, and a significant reduction, even at moderate NaCl concentrations (50 mM), was noticed. Conversely, the relative emission from PSI increased, and emission from uncoupled LHCs appeared in high-salt (≥100 mM NaCl) conditions. In the case of *stt7* cells, PSII emission was not reduced to the same extent as in WT, but *stt7* showed more significant amounts of LHCII aggregates in high-salinity media. These results suggest that (1) LHCII phosphorylation was the primary cause for the decreased PSII antenna size under salt stress, and (2) the phosphorylation-induced changes (in WT) protect against salt-induced degradation of the photosynthetic pigment–protein complexes.

### Time-resolved fluorescence

**Figure 4:**
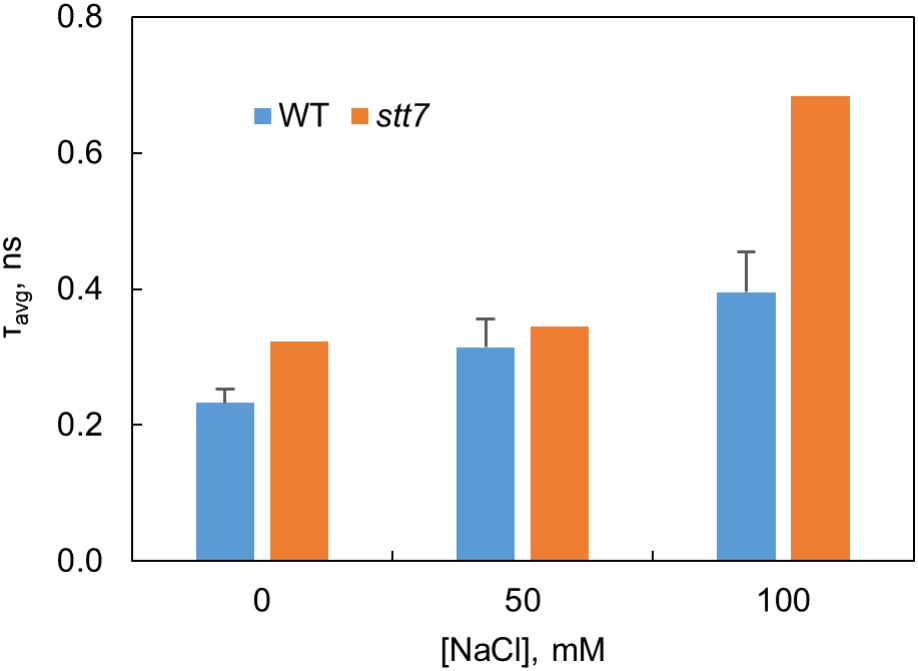
Average fluorescence decay lifetimes at 680 nm determined from the fluorescence kinetics of *C. reinhardtii* WT and state transition kinase 7 (*stt7)* cells grown in media of different salinity.

Fluorescence decays of dark-adapted WT and *stt7* cell suspensions were recorded with picosecond time resolution at emission wavelengths from 670 to 740 nm. The fluorescence decay kinetics can be described with several lifetimes—60 ps, primarily associated with PSI, and 0.2–1 ns that can be attributed to PSII (**Supplementary Figures S4A–D**). The slowest lifetime component (1.5–2 ns) reflects a small number of closed PSII RCs (Unlu et al., 2014). The PSII decay lifetimes in salt-grown cells were longer compared to the control, and a decay component with a 3–4-ns lifetime can be associated with the presence of energetically uncoupled pigments—in agreement with the emission spectra at 77 K (**Supplementary Figure S3**). As a result of these changes, the average fluorescence lifetimes in salt-treated cells increased (**Figure 4**), which indicates a reduced photochemical quantum yield of PSII. Based on this parameter, the *stt7* mutant also appeared more sensitive to high salinity than WT.

### Circular dichroism spectra

**Figure 5:**
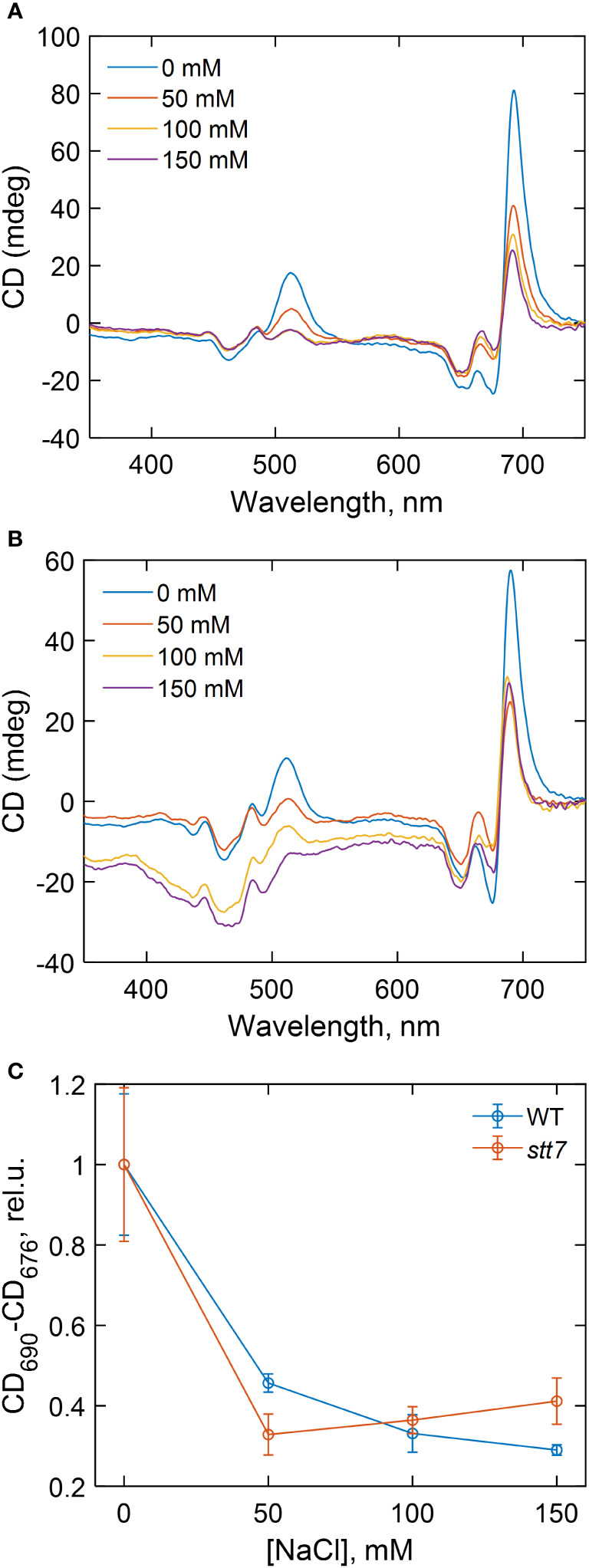
Circular dichroism spectra of **(A)** wild type (WT) and **(B)** state transition kinase 7 (*stt7) C. reinhardtii* cells grown under different salinity concentrations, as indicated, and **(C)** dependence of the total amplitude of the psi-type circular dichroism (CD) bands in the red spectral region (CD_690_-CD_676_) on the salinity of the growth medium.

Circular dichroism (CD) in the visible region originates primarily from electronic couplings (excitonic interactions) between pigments (Chls and carotenoids) in the thylakoid membranes and is very sensitive to changes in the structure and composition of the pigment–protein complexes and in the membrane macro-organization (Garab and van Amerongen, 2009). The highest-intensity bands in the CD spectra of intact cells (**Figure 5**), at (+) 510, (−) 676, and (+) 690 nm, termed as “psi-type” CD, originate from long-range interactions between many chromophores in chirally ordered macrodomains. The major psi-type bands diminished by 50%–70% in all strains grown even at moderate salt concentrations (50 mM), indicating that salinity induced significant alterations in the macro-organization of the thylakoid membranes (**Figure 5**). The magnitude of the salt effect on the psi-type CD was approximately equal in *stt7* as in WT (**Figures 5A, B**). At severe salt stress (150 mM), changes in psi-type CD bands were also accompanied by distortions of some excitonic bands in the Soret (430–490 nm) region, suggesting changes in the composition or molecular architecture of the pigment–protein complexes (**Supplementary Table S4**).

### Protein phosphorylation

**Figure 6:**
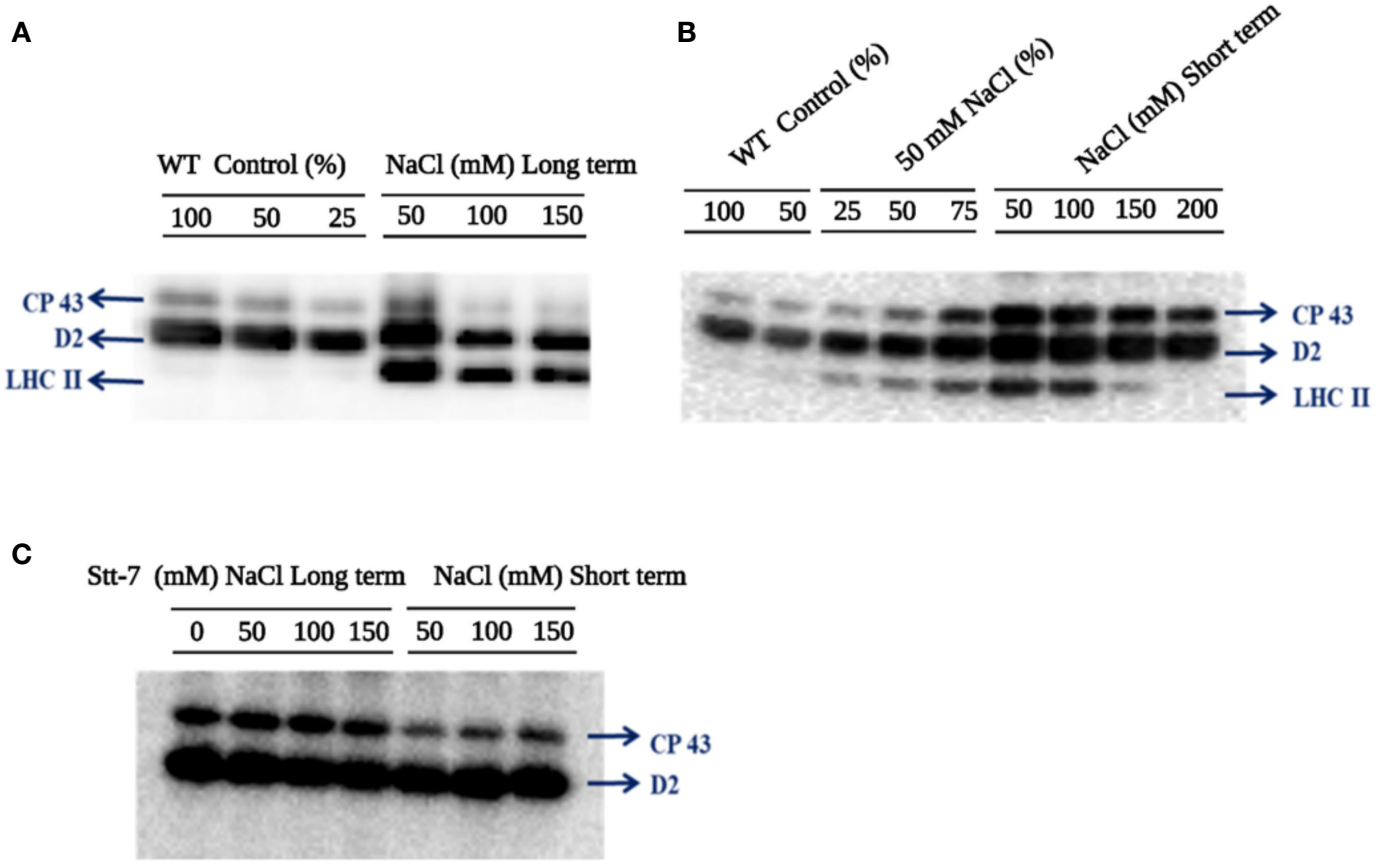
Immunoblotting analysis of thylakoid membranes probed with anti-phosphothreonine antibody **(A)** *C. reinhardtii* WT cells grown in various sodium chloride (NaCl) concentrations (0, 50, 100, and 150 mM for 96 hours (long-term). **(B)** *C. reinhardtii* WT control cells from the mid-log phase were treated with different NaCl concentrations (0, 50, 100, 150, and 200 mM) for 20 min (short-term). **(C)** *C. reinhardtii stt7* cells were grown in long-term (96 h) and short-term (20 min) conditions with the indicated NaCl concentrations. In order to compare the loading control, the wild-type samples were loaded with different dilutions (25, 50, and 100%).

The effect of NaCl on the phosphorylation status of thylakoid membrane proteins was studied in *C. reinhardtii* cells after long-term (4 days) and short-term (20 min) salt treatment. **Figure 6** shows immunoblot analysis of thylakoid proteins from cells grown in different NaCl concentrations (long- and short-term), probed with an anti-phosphothreonine antibody. Differential phosphorylation of CP43, D2, and LHCII subunits was observed in WT cells in both long- and short*-*term conditions. Surprisingly, LHCII subunits were phosphorylated in both types of treatment (**Figure 6A**). However, in short-term tests, the maximum phosphorylation level was attained at 50 mM NaCl and decreased with the increasing salt concentration. No LHCII phosphorylation was detected at 200 mM NaCl (**Figure 6B**). Phosphorylation of the PSII core proteins, CP43 and D2, also seemed to be maximal at 50 mM NaCl and further decreased at higher concentrations.

To confirm that LHCII phosphorylation in high-salt conditions was mediated by the Stt7 kinase involved in light-induced state transitions, we used the *stt7* mutant, which lacks the kinase. As expected, neither cells grown for 4 days in high salinity media nor after short-term salt treatment showed any LHCII phosphorylation (**Figure 6C**). Thus, we infer that the significant LHCII phosphorylation is due to NaCl-treatment-related Stt7 kinase activation. Hence, moderate (50 mM) salinity triggers the LHCII phosphorylation *via* the *Stt7* kinase activation observed in light-induced state transitions (Subramanyam et al., 2006).

### Thylakoid membrane ultrastructure

**Figure 7:**
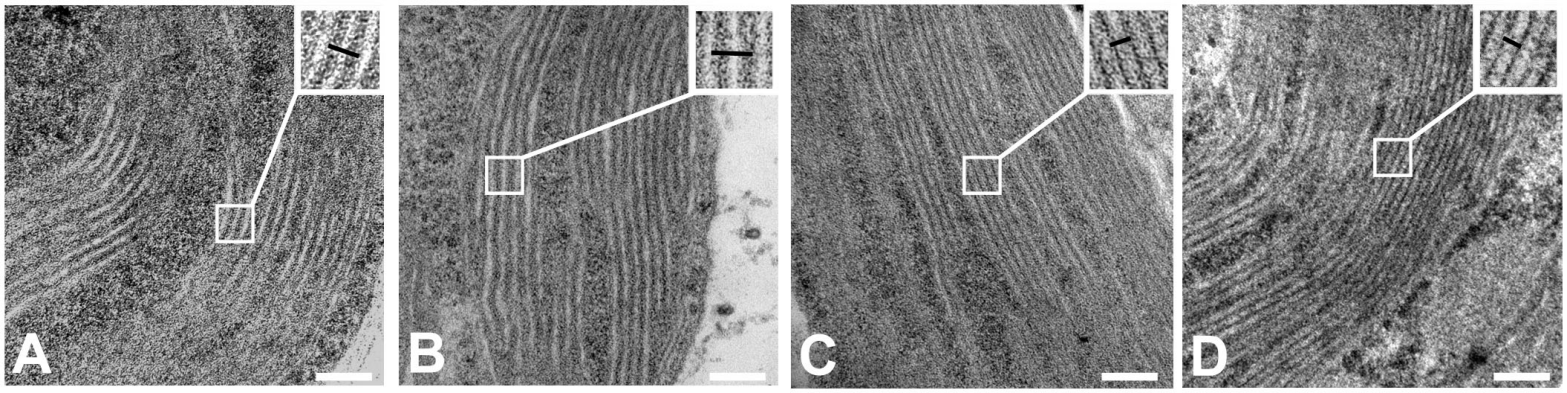
Transmission electron micrograph images of *C. reinhardtii* strains: **(A)** WT cells control, **(B)** WT cells grown in 100 mM sodium chloride (NaCl) containing media, **(C)** state transition kinase (*stt7)* cells control, and **(D)** *stt7* cells grown in 100 mM NaCl containing media (bars: 100 nm).

Transmission electron microscopy (TEM) showed that the typical stacked grana thylakoid membrane ultrastructure was retained in salt-grown cells (100 mM NaCl) (**Figure 7**). The repeat distance, i.e., the distance between two neighboring granal membranes or stromal gaps, reflects the degree of stacking or the level of appression of the granal thylakoid membranes. The repeat distances between thylakoid membrane layers increased by approximately 2 nm in salt-grown WT cells (**Supplementary Table S1**). To some extent, these ultrastructural changes are reminiscent of light-induced state transitions—swelling of the lumen, loosening of the grana stacks, and fragmentation of the granum bodies have been observed upon transition to State II in *A. thaliana* (Chuartzman et al., 2008). Correspondingly, there was no significant change in the repeat distances in salt-treated cells of the *stt7* mutant.

### Protein composition of PSI and PSII supercomplexes

**Figure 8:**
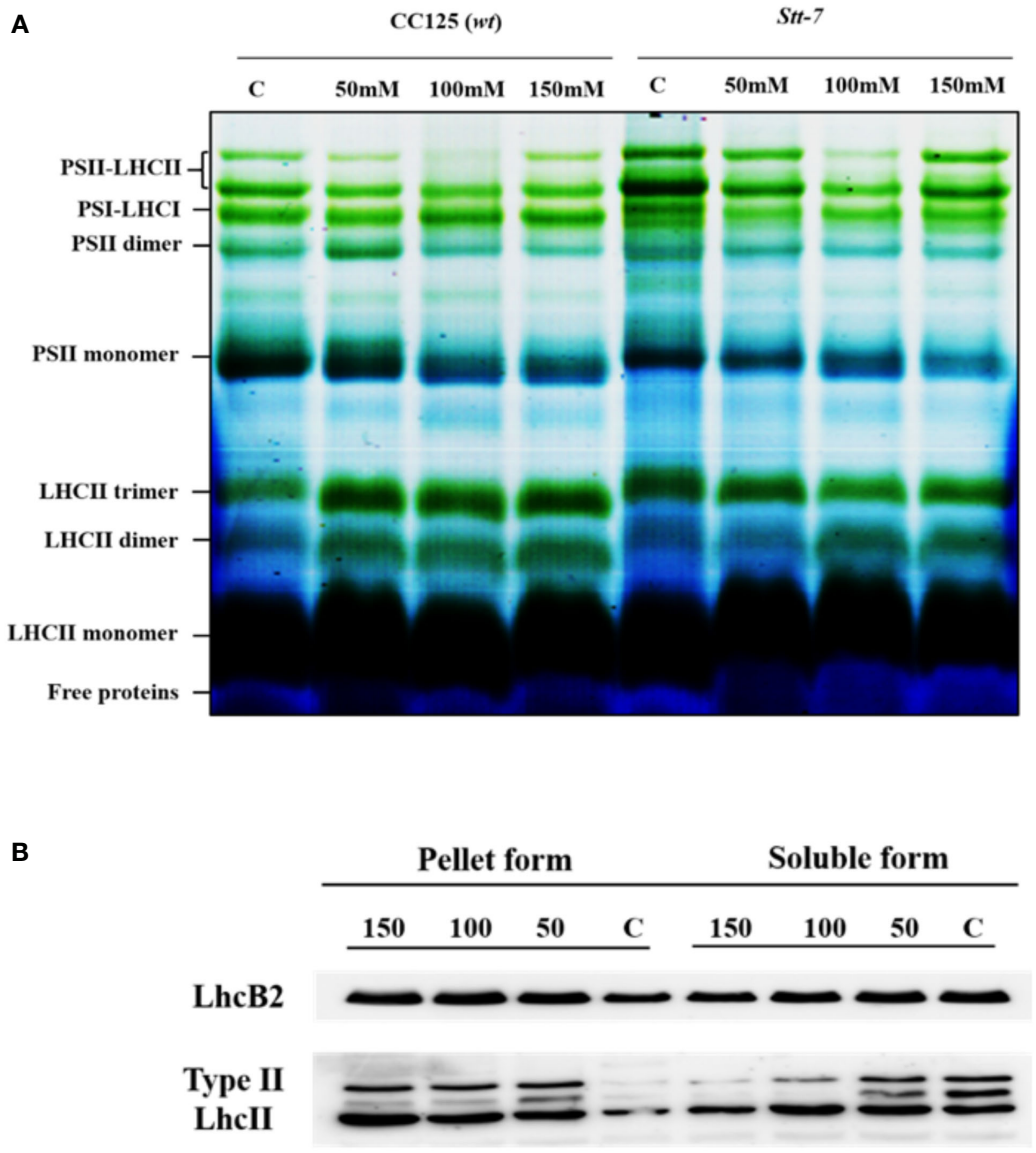
Blue native PAGE and Western blot from 0, 100, and 150 mM salt-treated conditions. **(A)** Blue-native gel electrophoresis of thylakoid membranes solubilized with 0.8% β-DM isolated from *C. reinhardtii* WT (CC125) and *stt7* cells grown in media with 0, 50, 100, and 150 mM NaCl. 8 µg of Chl was loaded into each lane. **(B)** Thylakoids isolated from 0-, 50-, 100-, and 150-mM sodium chloride treated cells solubilized with mild detergent 0.3% of α-DM + 0.5% of Digitonin based on equal Chl (30 µg). The soluble form (20 µl) was collected for SDS gel electrophoresis. The remaining insoluble pellet was solubilized with SDS sample buffer containing 0.1 M dithiothreitol (DTT), 10% SDS, 0.5M Tris in control as well as (50, 100, and 150) mM salt-treated samples which were fractionated by SDS-PAGE to demonstrate the appearance of Lhcb2 and Lhcbm5.

To characterize the organizational changes in PSI and PSII supercomplexes from cells grown under salt stress, we analyzed the protein composition of isolated thylakoid membranes by two-dimensional PAGE. Protein complexes were first separated by blue-native (BN) PAGE (**Figure 8**), followed by SDS-PAGE as a second dimension (**Supplementary Figure S5A**). We identified a significant increase in the content of LHCII trimers in cells grown in high-salinity media, particularly 150 mM salt (**Figure 8A**). In contrast, no increase in the intensity of the LHCII trimer band was visible in *stt7* (**Figure 8A**). We used the Lhcb2 antibody to confirm the increased accumulation of LHCII trimers under salt stress in WT (**Figure 8B** and **Supplementary Figure S5A**).

On the other hand, the relative abundance of PSI and PSII core complexes was slightly lower (**Figure 8A**). Furthermore, we did not observe any significant changes in the pigment–protein complexes or protein levels in the second dimension except for a slight increase in the content of LHCII trimers (**Supplementary Figure S5B**). Thus, the detected changes are presumably part of the long-term adaptation mechanism.

Previous reports on nutrient stress and other reports on high-light stress have shown irreversible aggregation of light-harvesting complexes in higher plants (Yamamoto, 2001; Devadasu et al., 2021). To determine if the protein aggregation also occurred under salt stress, we did mild digitonin treatment and low-speed centrifugation of the thylakoid membranes, as suggested earlier (Yamamoto et al., 2008). Western blots showed that Lhcb2 and type II LHCII proteins accumulated in the pellet after salt treatment, whereas their concentration in the soluble fraction diminished (**Figure 8B**). These results support that LHCII aggregated under salt stress in *C*. *reinhardtii*.

### The protein content of the thylakoid membranes

**Figure 9:**
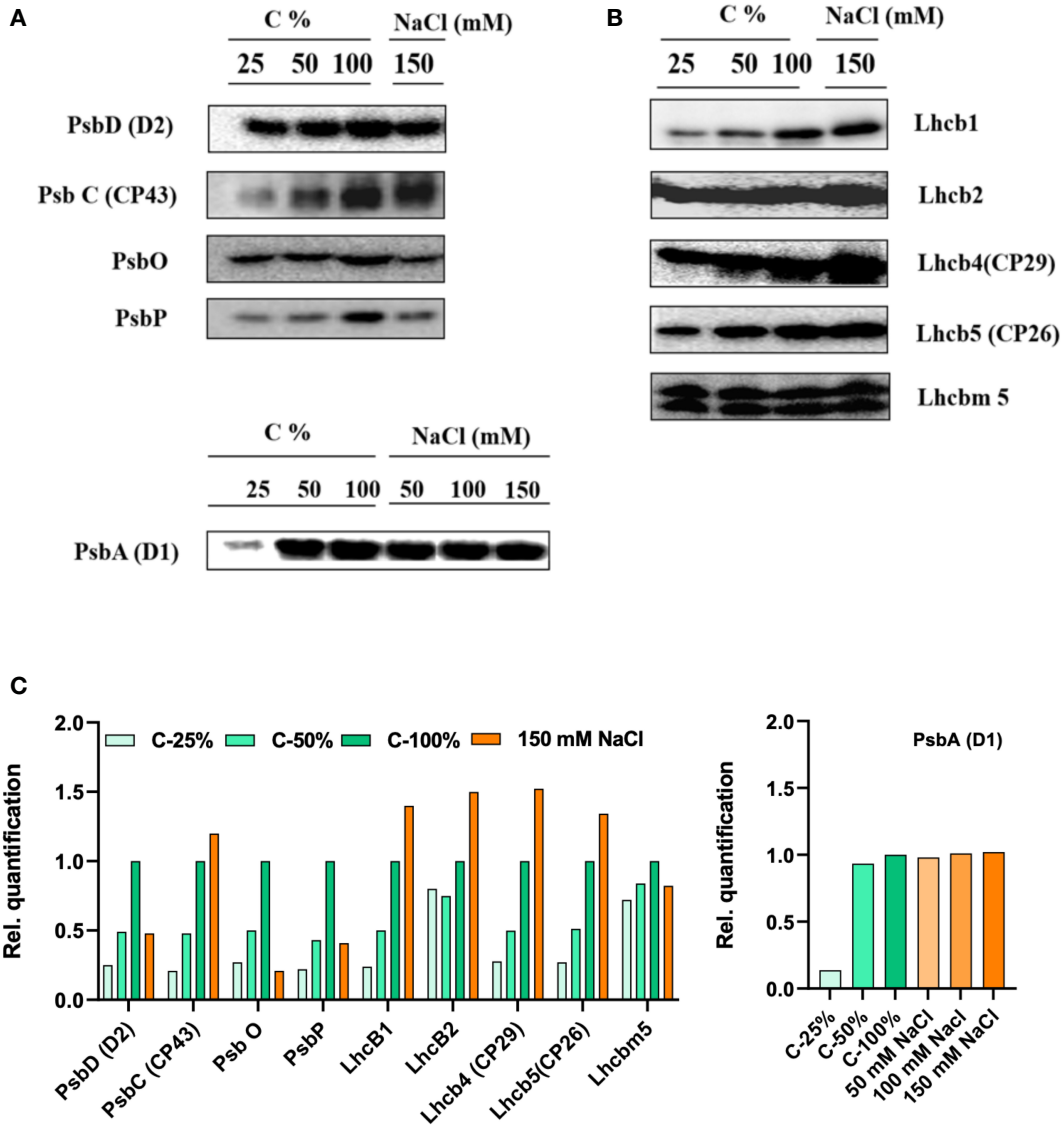
Protein profile analysis of thylakoid membranes isolated from 0 and 150 mM sodium chloride (NaCl) grown C. reinhardtii cells, probed with **(A)** photosystem (PS)II core antibodies (PsbD—D2, PsbC—CP43, PsbO, and PsbP) and with **(B)** LHCII antibodies (Lhcb1, Lhcb2, Lhcb4, Lhcb5, and Lhcbm5) **(C)** the relative density of proteins were quantified using ImageJ software. In order to compare the loading control, the wild-type samples were loaded with different dilutions (25, 50, and 100%).

**Figure 10:**
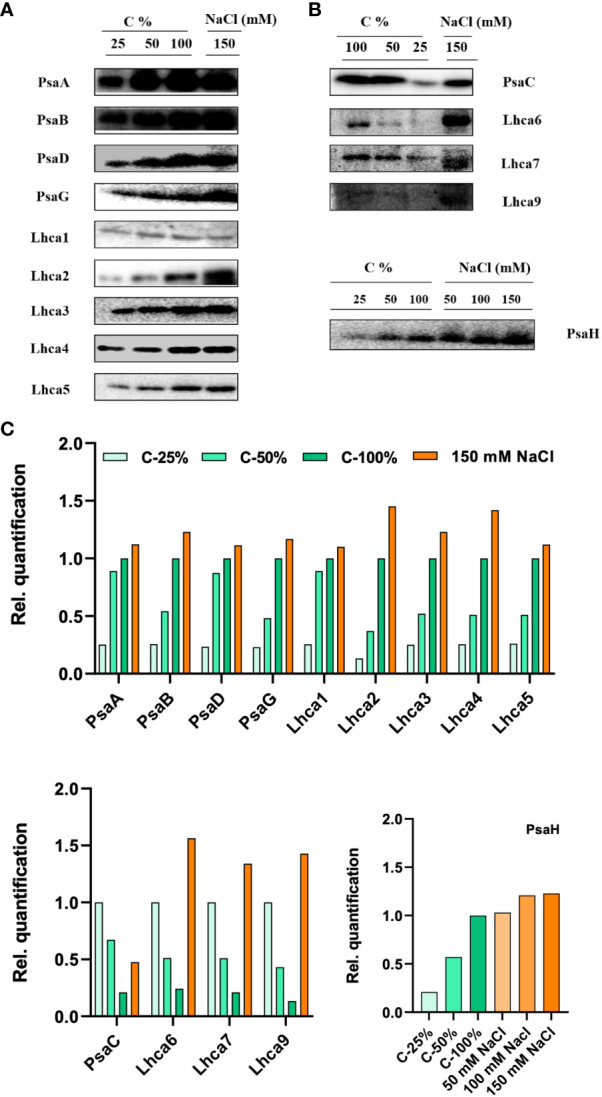
Protein profile analysis of thylakoid membranes isolated from control and 150 mM sodium chloride (NaCl) treated C. reinhardtii cells, **(A)** probed with photosystem (PS)I core antibodies (PsaA, PsaB, PsaC, PsaD, PsaG, and PsaH) and **(B)** LHCI complex proteins, (Lhca1, Lhca2, Lhca3, Lhca4, Lhca5, Lhca6, Lhca7, and Lhca9). Two micrograms of Chl was loaded on to each well. **(C)** Relative band density from each lane was calculated by ImageJ software. In order to compare the loading control, the wild-type samples were loaded with different dilutions (25, 50, and 100%).

The thylakoid protein profile of cells grown in different salt concentrations (0–150 mM) was quantitatively determined by immunoblotting with specific antibodies against PSII-LHCII (**Figure 9**) and PSI-LHCI (**Figure 10**) subunits. All tested PSII core complex proteins were significantly reduced in high-salinity media. The abundance of the PSII core complex polypeptides (D1, D2, and CP43) and the oxygen-evolving complex proteins (PsbO and PsbP) was reduced by 30%–50% in the control (at 150 mM NaCl). In contrast to the PSII core proteins, the abundance of LHCII proteins increased in high-salinity media, corroborating the native PAGE data.

Long-term salt stress led to a significant increase (more than twofold) in the levels of the PSI proteins PsaB, PsaG, and PsaH (**Figure 10**). We further checked the protein levels of PsaH and PsaG in *stt7*. PsaG levels were stable (no increase) in control and *stt7* in150 mM NaCl. LHCI protein levels increased in WT cells grown in 150 mM NaCl (**Figure 10**). Similar results were obtained in both cases by loading equal amounts of protein on the gels (**Supplementary Figure S5B**).

### Gene expression levels

**Figure 11:**
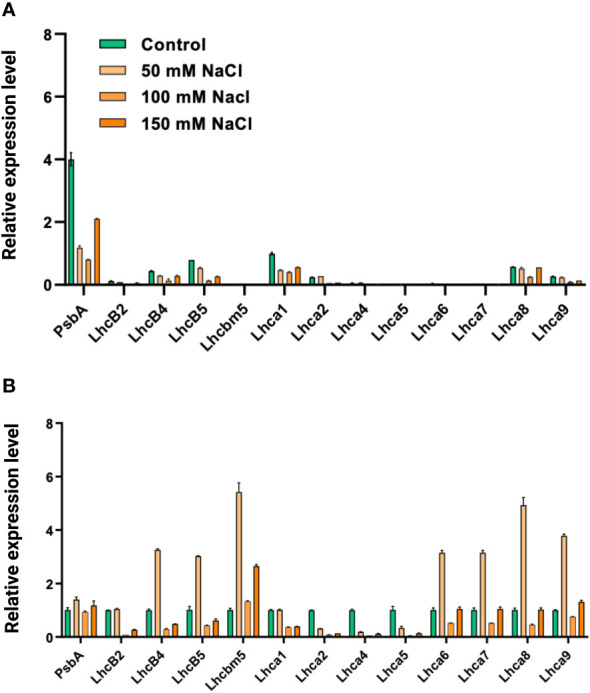
**(A)** The relative abundance of mRNA levels of genes from *C. reinhardtii* (CC125) and **(B)** state transition kinase mutant, (*stt7)* cells grown under 0, 50, 100, and 150 mM sodium chloride (NaCl) conditions., PSII gene (*psbA*), LHCII genes (*lhcb2, lhcb4, lhcb5*, and *lhcbm5*) and LHCI genes, (*lhca1*, *lhca2*, *lhca4*, *lhca5*, *lhca6*, *lhca7*, *lhca8*, and *lhca9)* were monitored under same salt stress condition. All the genes were normalized with the housekeeping gene, Histone H3.

We tested the levels of expression of the *psbA* gene, which encodes the D1 subunit of the PSII core complex, and several genes encoding light-harvesting complexes (LHCII genes—*lhcb2*, *lhcb4*, *lhcb5*, and *lhcbm5*; LHCI genes—*lhca1–2*, *lhca4–9*) in salt-grown WT and *stt7* cells (**Figure 11**). The transcript levels of LHCII and LHCI genes were reduced about twofold under salt stress in both WT and *stt7*. A similar trend was observed with the *psbA* gene under salt stress with WT (**Figure 11A**). In contrast, no reduction in *psbA* gene expression was found in the *stt7* mutant, suggesting a link between the salt-induced phosphorylation and downregulation of the PSII core complex content (**Figure 11B**). The transcript levels indicate an overall reduction in photosynthetic proteins in the cells and do not reflect their relative content on a Chl basis.

## Discussion

### Palmelloid formation as a stress response

Our previous studies showed that high salt concentrations affected the PSI and PSII proteins and their functions; however, the organization and acclimation have not been studied. Several studies have shown that *C. reinhardtii* transforms into small, somewhat ordered clumps termed palmelloids. The gradual loss of synchrony in cell division was visible from the palmelloids (**Figure 1A**), growth curves, and cell division time (6–8 h) (Neelam and Subramanyam, 2013). Palmelloid phenotype is a plastic response that certain stimuli can trigger. Various reasons have been described for the formation of these palmelloids in *C. reinhardtii*, as a defense mechanism in biotic stress (Lurling and Beekman, 2006), nutrient deprivation (Moulton and Bell, 2013), flagellar malfunction, cell wall abnormality, and pH changes in the environment (Neelam and Subramanyam, 2013)—all being the adaptive mechanisms of the organism to survive unfavorable conditions. There could be several reasons for the multicellular response in palmelloids: 1) cells might have increased the duration/frequency of cell division, 2) cells are stickier and tend to cohere, or 3) individuals exist in a paired or multicellular state (Moulton and Bell, 2013). We can eliminate the latter two possibilities, as *C. reinhardtii* is a unicellular organism and cells do not stick to each other. We presume that the cell wall is the main reason for these palmelloid formations and have chosen cell-wall-deficient (CC)-503, a class III cell wall mutant. The absence of palmelloids in the CC-503 mutant corroborated our hypothesis that palmelloid formation under salt stress is due to alterations in the cell wall. In addition, this mutant exhibits the normal flagellar motility. Hence, it appears that in the WT after cell division, daughter cells are locked inside the mother cell wall. Apart from NaCl, other salts like KCl and CH_3_COOK also induced palmelloid formation in *C. reinhardtii* (**Figures 1A–D**). Furthermore, we performed biochemical characterizations in both strains to identify the reasons behind this salt tolerance. No remarkable changes in the OJIP transients (**Supplementary Figure S6A**), absorption spectra, or protein profile of WT CC-125 and CC-503 were noticed (**Supplementary Figure S6B**).

### Inhibition of photosynthesis

*C. reinhardtii* displayed a shock response when treated with 150 mM NaCl for 1 h (Mastrobuoni et al., 2012). In addition, electron transport inhibition at donor and acceptor sides of PSII, with a shift in excitation energy distribution in favor of PSI, has been reported in cyanobacterial *Arhtrospira platensis* cells grown in 0.8 M NaCl for 12 h (Lu and Vonshak, 2002). In another report, incubation of *Synechococcus* sp. PCC7942 treated with NaCl reduced oxygen evolution activity in 1.5 h and led to a loss of PSI activity (Allakhverdiev et al., 2000).

The photosynthetic performance of salt-treated cells in our experiments was also impaired. We observed a decrease in electron transport and PSII photochemical efficiency (as evidenced by reduced oxygen evolution activity) and reduced levels of PSII core proteins, including the extrinsic subunits of the oxygen-evolving machinery (PsbO, PsbP), paralleled by reduced oxygen-evolution activity (Neelam and Subramanyam, 2013). Interestingly, the efficiency of photosynthesis in *stt7* was reduced drastically compared to the WT due to the absence of Stt7 kinase; hence, *stt7* cannot acclimate to the salt stress (**Supplementary Figure S2C**).

### Salt stress triggers Stt7-mediated LHCII phosphorylation

From the phosphorylation data, it was evident that NaCl triggered LHCII phosphorylation in both long-term (salt-grown cells) and short-term NaCl-exposed cells of *C. reinhardtii*. It is interesting to confirm that NaCl triggers state transitions as a stress response reminiscent of light-induced state transitions in terms of LHCII phosphorylation (**Figure 6**). Similar temperature-induced-state transitions have been reported in *A. thaliana* (Nellaepalli et al., 2011; Nellaepalli et al., 2015) and *C*. *reinhardtii* (Madireddi et al., 2019). Surprisingly, LHCII phosphorylation was also observed in long-term salt treatments (cells grown in 100 mM and 150 mM salt concentrations). State transitions have been regarded as a short-term response to balance the energy between photosystems, PSII and PSI. Several publications reported that the PQ pool becomes reduced under low light exposure for a short duration, and LHCII gets phosphorylated by the Stt7 kinase enzyme in *C. reinhardtii*. Furthermore, the P-LHCII migrates to PSI and makes PSI–LHCI–LHCII complexes (Subramanyam et al., 2006; Nagy et al., 2014). A comparison with the *stt7* mutant confirmed that LHCII phosphorylation under short- and long-term salt stress was mediated by the same *Stt7* kinase involved in light-induced state transitions. It was reported that stress factors light and temperature could trigger STN7 kinase activation in *A. thaliana* (Nellaepalli et al., 2011; Nellaepalli et al., 2012; Ghysels et al., 2013). The phosphorylation of LHCII could be involved in the reorganization of supercomplexes to protect against salt stress.

Long-term salt treatment of cells resulted in significant changes in the protein composition of PSI and PSII. Salt-treated cells contained substantially higher amounts of LHCII and LHCI at the expense of PSII and PSI core proteins, respectively (**Figures 9**, **10**). These changes, together with the Stt7-dependent LHCII phosphorylation, could be triggered by persistently reduced PQ pool under high salinity conditions. A reduced state of the PQ pools can also explain the increase in F_o_ fluorescence level in high salt conditions. A reduced PQ pool activates the Stt7 kinase in the short term and promotes a shift in photosystem stoichiometry in the longer term to restore the balanced energy flow (Anderson et al., 1995; Montané et al., 1998).

### Moderate salt treatment disrupts the membrane macro-organization

From the circular dichroism data, it was evident that even moderate salt stress (50 mM NaCl) had a significant effect on the macro-organization of protein complexes in the thylakoid membranes. In addition, severe salt stress was further accompanied by changes in the composition of the pigment–protein complexes. As part of acclimatization responses to the changing environmental conditions, photosynthetic supercomplexes undergo supramolecular reorganizations like state transitions and non-photochemical quenching (Minagawa, 2013). Phosphorylation of LHCII can interfere with protein packing, specifically with the ordered arrangement of PSII–LHCII supercomplexes in the membrane (Boekema et al., 2000; Allen and Forsberg, 2001), and interrupt the close packing of grana stacks—both of these phenomena are sensed by the psi-type CD (Garab and van Amerongen, 2009). However, the observed decrease in the psi-type CD signal appears to be a direct effect of the NaCl treatment, irrespective of LHCII phosphorylation.

The phosphorylation of LHCII in salt-grown cells (but not in *stt7* mutant) can be correlated with the thickening or swelling of the grana thylakoids. We can postulate that the changes in the thylakoid stacking are mediated by P-LHCII, similarly to the significant structural rearrangements associated with light-induced state transitions in higher plants (Chuartzman et al., 2008), where P-LHCII alters the grana stacking, grana-stroma interconnections, and the composition of grana and stroma lamellae, in which lhcb1 and lhcb2 were involved in phosphorylation (Chuartzman et al., 2008).

Cruz et al. (2001) reported significant changes in the thylakoid membrane ultrastructure when *C. reinhardtii* cells were subjected to hyperosmotic shock—severe disruption of the grana stacking and shrinkage of the lumen, which was correlated with a slowdown of intersystem electron transfer, presumably because of the hampered diffusion of plastocyanin. In our experimental conditions, the changes in the thylakoid macro-organization are comparatively milder, and we did not observe significant lumen shrinkage in the electron micrographs (**Figure 7**). Nevertheless, the salt-induced functional changes detected by fluorescence might be partly caused by the changes in membrane macro-organization.

### Changes in the photosystem antenna size

In light-induced state transitions, the fluorescence emission spectra of salt-treated *C. reinhardtii* cells showed a pronounced decrease in PSII emission in favor of PSI emission even at moderate NaCl concentrations (50 mM). This effect was not observed in the *stt7* mutant unable to phosphorylate LHCII. However, in high-salt conditions, the emission spectra indicated the formation of LHCII aggregates (**Supplementary Figure S3**). Mild solubilization of the thylakoid membranes further indicated that LHCII proteins were aggregated under high-salt conditions (**Figure 8B**). Furthermore, the enhanced 678 nm intensity in the low-temperature emission spectra and the appearance of long (nanoseconds) decay components in the fluorescence decay at high salinity likely originate from a small fraction of energetically detached LHCs (**Supplementary Figure S7**). This also corroborates the more reduced PQ pool, higher F_o_ level observed in the fluorescence induction transients, and the reduced maximal variable fluorescence (F_v_/F_m_, **Supplementary Figure S2**). Uncoupling of LHCs also occurs in the absence of phosphorylation (in the stt7 mutant)—even more so than in WT at high salt concentration. The more severe salt-induced effects (on the fluorescence kinetics and 77 K emission spectra, **Figure 2**) hint that LHCII phosphorylation, in this case, is an acclimatory stress response stabilizing the photosynthetic apparatus.

### Remodeling of photosystems

The differential response of PSI and PSII core protein subunit composition indicates that salt stress may change the photosystem supercomplexes. The H subunit of PSI is known to be involved in docking phosphorylated LHCII in state transitions (Lunde et al., 2000). It can be speculated that the salt-induced increase in the PsaH level, a subunit of PSI, observed in WT but not in the *stt7* mutant, is part of the Stt7-dependent adaptation mechanism. The levels of the PsaG protein were increased in salt-grown (150 mM) cells in the WT strain; however, unaltered levels of this protein in the mutants could explain its role in tuning the PSI activity. A study of *Arabidopsis* mutants (ΔPsaG) has shown the high amounts of poorly connected LHCI and increased NADP+ photoreduction rate compared with the wild type (Jensen et al., 2002; Varotto et al., 2002). Our previous reports show that salt also affects PsaE (Subramanyam et al., 2010). It is known that the PsaE protein is located at the reducing side of PSI and stabilizes the stromal hump, which is involved in docking soluble electron acceptors like ferredoxin/flavodoxin (Lelong et al., 1996).

We assume that salt-grown *C. reinhardtii* cells acclimatize to remodeling the PSI and PSII proteins through the Stt7 kinase. It was reported that LHCI composition/stoichiometry varies in response to changing environmental conditions; moreover, LHCI in *C. reinhardtii* is larger than plant LHCI and helps in light-harvesting along with photoprotection (Bailey et al., 2001; Ben-Shem et al., 2003). Another report indicated that Lhca3 played a significant role when LHCI was remodeled and demonstrated the upregulation of Lhca4 and 9 in Fe-deficiency cells from *C. reinhardtii* (Naumann et al., 2005). In addition, a recent report from *C. merolae* grown under a broad range of temperatures revealed that remodeling processes of photosynthetic proteins tuned photosynthesis according to the demands placed on the system (Nikolova et al., 2017). However, the lower levels of transcripts and increased content of LHC proteins suggested changes in cellular physiology and metabolism to acclimatize to high-salt conditions. Thus, the short- and long-term salt response in *C. reinhardtii* is related to Stt7-dependent LHCII phosphorylation, which facilitates the acclimation process by remodeling the photosynthetic proteins.

## Concluding remarks

The results show that moderate salt stress (50 mM NaCl) elicits various structural and functional responses in *C. reinhardtii* at the level of the thylakoid membranes and photosynthetic proteins. The most significant observation is the induced accumulation of LHCII, the Stt7*-*dependent LHCII phosphorylation, and the resulting changes in the membrane ultrastructure, photosystem composition, and excitation energy distribution. Several lines of evidence point to a higher sensitivity of the photosynthetic apparatus to high salinity in the *stt7* mutants. These data suggest that the salt-induced “state transition,” i.e., Stt7-kinase-dependent LHCII phosphorylation, could be an acclimatory response; however, further experiments would be needed to evaluate the extent of protection that it may endow against high-salinity damage to the photosynthetic apparatus.

## Data availability statement

The raw data supporting the conclusions of this article will be made available by the authors, without undue reservation. Additional information can be seen in **Supplementary Figures S1–S7** and **Supplementary Tables 1–4**.

## Author contributions

SBN, BU, GG, PL, and RS conceived, designed, and performed experiments and data analysis. ED, SBN, SK, RY, SN, PA, TP, and BU performed experiments. SBN, GG, PL, and RS conceived the experiments and participated in experimental design and data interpretation. SBN, GG, PL, and RS co-wrote the paper. All authors contributed to the article and approved the submitted version.
