# Supplementary material for: Long- and short-term acclimation of the photosynthetic apparatus to salinity in Chlamydomonas reinhardtii. The role of Stt7 protein kinase
Source: Front Plant Sci. 2023 Apr 5;14:1051711. doi: 10.3389/fpls.2023.1051711 (PMC10113551; doi:10.3389/fpls.2023.1051711)
Supplement: Supplementary file 1 [file DataSheet_1.docx]

**Supplemental Information**


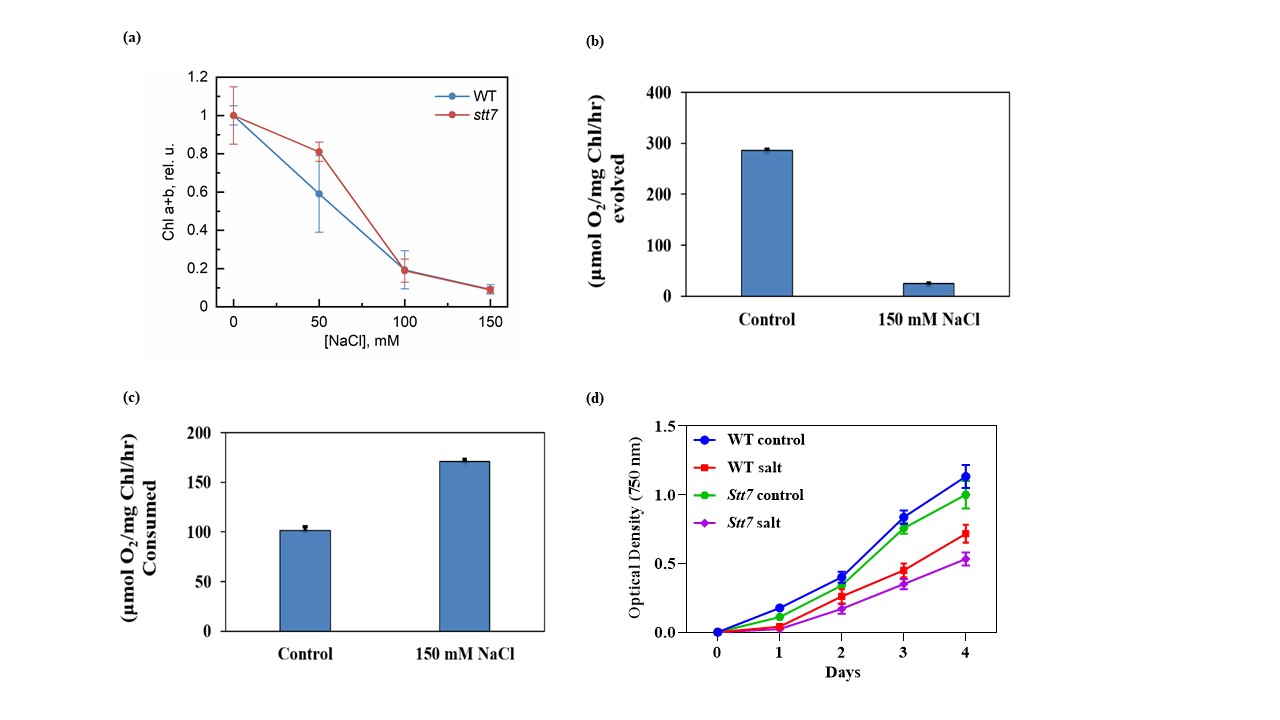


**Figure S1.** (a) Changes in total chlorophyll content (calculated in µg/ml) of WT and *stt7 C. reinhardtii* cells grown under different concentrations of NaCl, relative to the Control (0 mM NaCl). **(**b**)** Oxygen evolution of whole cells and (c) oxygen uptake activity of isolated thylakoid membranes of *C. reinhardtii* cells grown in media containing different concentrations of NaCl: 0 (control) and 150 mM, (d) Growth curve of WT and *stt7* in control and 150 mM salt condition.


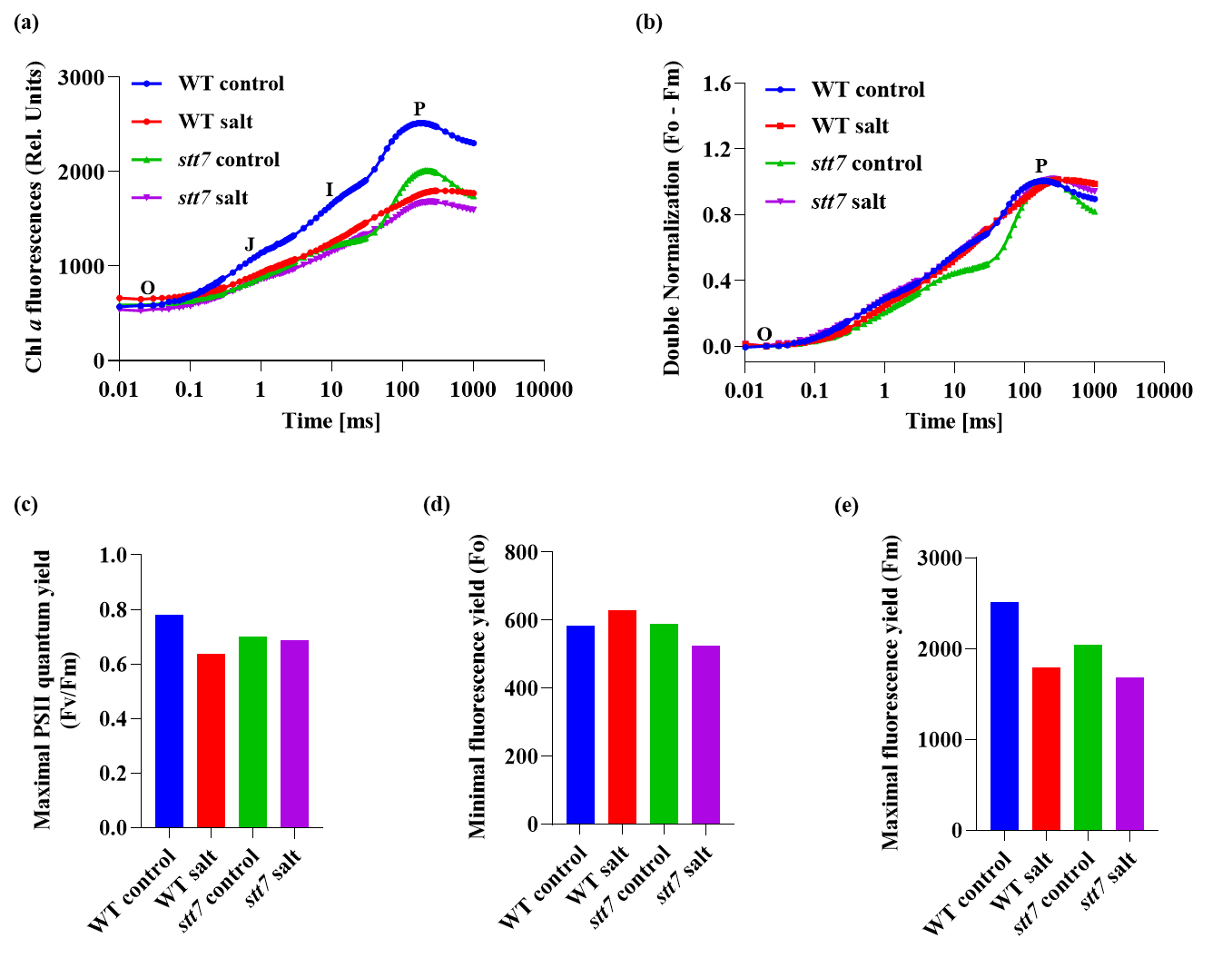


**Figure S2.** (a) Chlorophyll *a* fluorescence transient of WT and *stt7 C. reinhardtii* cells grown in media containing 0 mM and 150mM NaCl. (b) Double normalized chl *a* fluorescence kinetics from F_o_ to F_m_, (c) Maximum PSII quantum yield (F_v_/F_m_), (d) Minimum fluorescence yield (F_o_) and (e) Maximum Fluorescence yield (F_m_) of WT and *stt7* in control and 150 mM salt condition.

**Figure S3.** Gaussian-component decomposition of the 77 K fluorescence emission spectra. (a) WT control; (b) WT cells grown in a medium containing 100 mM NaCl.

**Figure S4.** Decay-associated spectra of (a), (b) wild-type and (c), (d) *stt7* cells of *C*. *reinhardtii*. (a), (c) control and (b), (d) 100 mM NaCl treated cells.


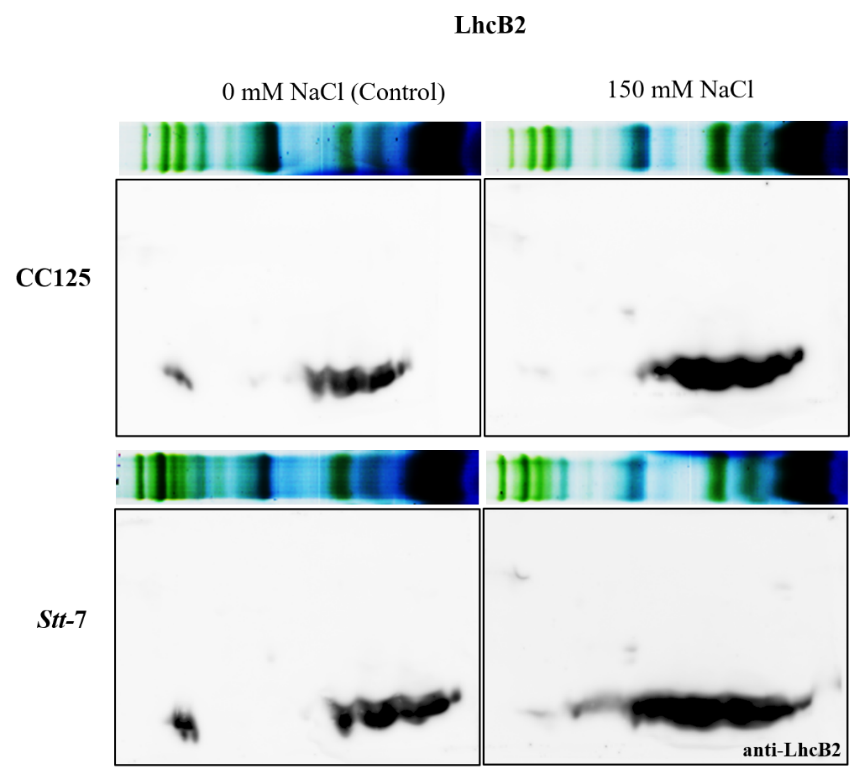


(a)

(b)


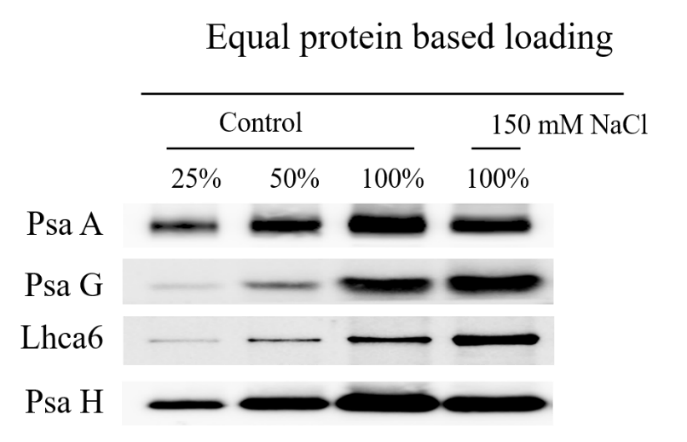


**Figure S5**. The blue native gels were run on the second dimension using SDS-PAGE; a). The lanes were carefully dissected, denatured and placed on 15% Bis-Tris gel. After running the gel, it transferred to nitrocellulose membrane and probed with Lhcb2 antibody to check the abundance of LHCII complex during the salinity stress; b) Western blots showing the equal protein-based loading (2 µg of protein per lane were loaded). We were detected with specific antibodies (a) PsaA**, (**b) PsaG, (c) Lhca6, (d) PsaH. Experiments were done in three biological replicates, and we observed similar results. Control samples were loaded based on percentages (25%, 50%, and 100%) wise for comparison.


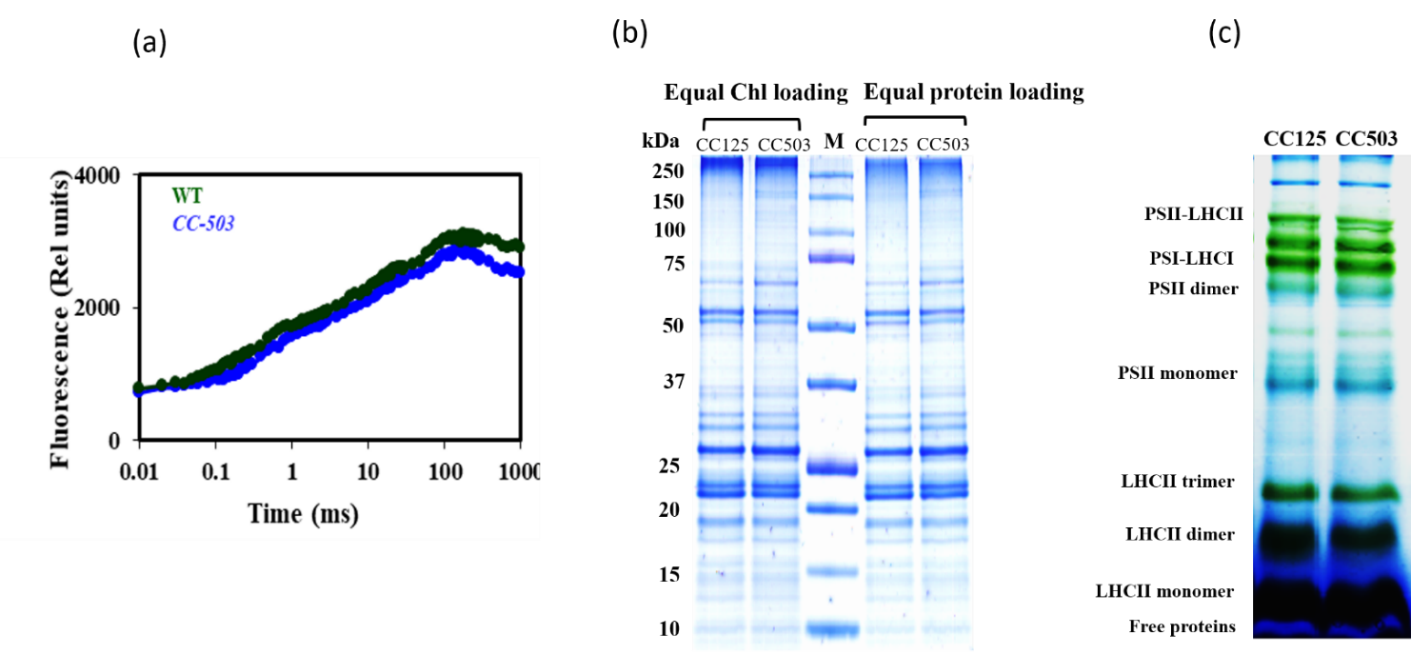


**Figure S6.** Characterization of both WT (CC125) and CC-503 strains. (a) Chl *a* OJIP fluorescence curves were obtained from Handy PEA instrument, (b) Denaturing SDS PAGE Protein profile was characterized with an equal amount of protein (2 µg per lane) as well as chlorophyll (1µg per lane), (c) membrane organization of thylakoids isolated from WT and CC-503 strains in the Blue native PAGE and 7 µg of Chl was loaded to each lane after solubilization with β-DM (0.8%).


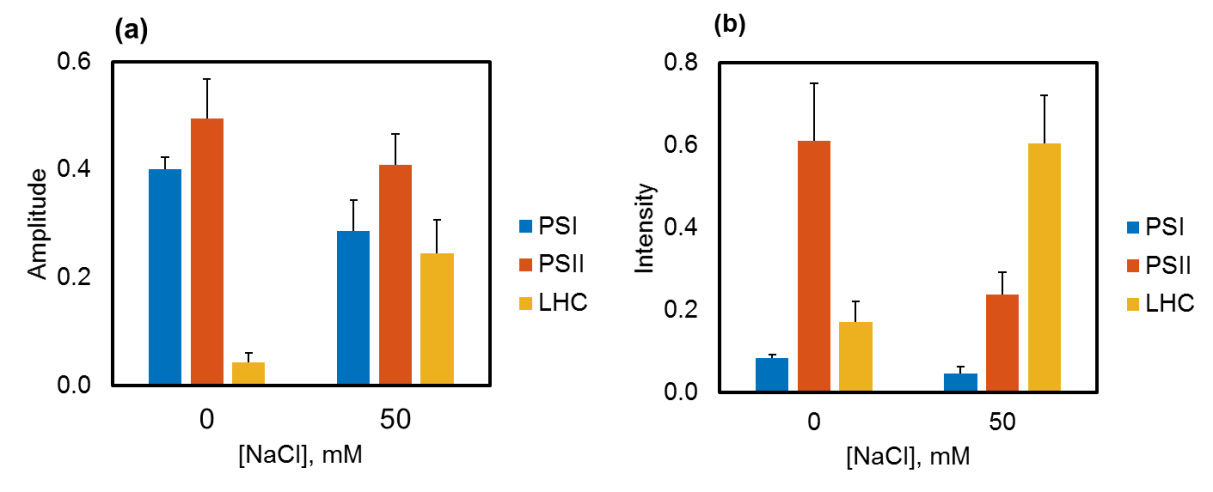


**Figure S7.** (a) Relative amplitudes and (b) relative fluorescence intensities (amplitude * lifetime) of the fluorescence lifetime components associated with PSI (60-70 ps lifetime), PSII (200-700 ps lifetimes) and free LHC (lifetimes > 1 ns). The values are average from 4 independent experiments; error bars show standard errors.

**Supplementary Table** 1**.** Thylakoid repeat distances (nm) of control and salt (100 mM NaCl) grown cells of WT and *stt7* measured on transmission electron micrographs. The data represent mean ± SE of three different batches.

| **Repeat distance (nm)** | | | |
| --- | --- | --- | --- |
| **WT control** | **WT salt** | ***stt7* control** | ***stt7* salt** |
| 21.885 ± 0.33 | 23.7 ± 0.59 | 20.375 ± 0.31 | 20.725 ± 0.42 |

**Supplementary Table 2.** List of primers used for RT-PCR analysis from salinity conditions.

| Gene | Forward primer (F) 5ʹ - 3ʹ | Reverse primer (R) 3ʹ - 5ʹ | Id |
| --- | --- | --- | --- |
| *Lhca1* | GCGAGGTCATCCACGGTCG | CCAGCAGGGCGTTCAGGTC | Cre06.g283050 .t1.2 |
| *Lhca2* | GGAGGGCAAGCGCTACGAG | GCCCTTGCCGTACACAGCG | Cre12.g508750 .t1.2 |
| *Lhca3* | CCAGATGGGCCTGATGGGGA | GGTTGAACATCAGCCCGCCG | Cre10.g454734 .t1.1 |
| *Lhca4* | CCATCTTCACCAACAACAAGCTGCC | CCGATGAAGCCAGCGAACGC | Cre10.g452050 .t1.2 |
| *Lhca5* | GGTGCCCAACCCGGAGATG | GGATGAAGGCCATGTAGGCGATCA | Cre10.g425900 .t1.2 |
| *Lhca6* | CGGTATCCTGGTGCAGGAGATCG | GGCTGAAGATGGGGTCCTGGT | Cre06.g278213.t1.1 |
| *Lhca7* | GGCAAGGTGGTCGTGGAGAAG | CCGTTCTCCAGGCCCTTGAACTC | Cre16.g687900.t1.2 |
| *Lhca8* | GCCTGAGTGGTACGACGCC | CCTCGAAGCCCAGGAACGAG | Cre06.g272650.t1.2 |
| *Lhca9* | GGAGGCTGGCGCTAAGGAG | GGGAAGGAGTTGATGAAGCCGG | Cre07.g344950.t1.2 |
| *Psb A* | GTATTTGGTTCACTGCTTTAG | GGAAGTTGTGAGCGTTACGC | Cre.cp2716987 |
| *LhcB2* | CGTTCGGTGAGGCTGTCTGGTTC | CCTCAGCCAGGCCCATCAC | Cre12.g548400 .t1.2 |
| *LhcB4* | GCCCTTCTCCATCACCCAGC | CGTCCTCAGAGGCCAGCTTGAG | Cre17.g720250 .t1.2 |
| *LhcB5* | CGGGCATTGGCAAGTTCGACTC | GCACGGAGACCATGGCCAG | Cre16.g673650 .t1.1 |
| *LhcBM5* | CGCTATTGAGGGCTACCGCGTC | CCAGGCGGCCGTTCTTGATC | Cre03.g156900 .t1.2 |

**Supplementary Table 3.** One-way ANOVA of the dependence of the fluorescence emission components on salt concentration (given in mM NaCl).

| **Component** | **WT** | | | ***stt7*** | | |
| --- | --- | --- | --- | --- | --- | --- |
|  | 50 | 100 | 150 | 50 | 100 | 150 |
| F676 |  |  | + |  |  | + |
| F685 + F694 | + | + | + |  |  | + |
| F700 |  |  | + |  |  | + |
| F710 |  |  |  |  |  | + |

**Supplementary Table 4.** One-way ANOVA of the dependence of the psi-type CD on salt concentration (given in mM NaCl).

| **NaCl (mM)** | **WT** | ***stt7*** |
| --- | --- | --- |
| 50 |  | + |
| 100 | + | + |
| 150 | + | + |
